# Collective Rabi-Driven Vibrational Activation in Molecular Polaritons

**DOI:** 10.1021/acs.nanolett.6c00832

**Published:** 2026-04-07

**Authors:** Carlos M. Bustamante, Franco P. Bonafé, Richard Richardson, Michael Ruggenthaler, Wenxiang Ying, Abraham Nitzan, Maxim Sukharev, Angel Rubio

**Affiliations:** † Max Planck Institute for the Structure and Dynamics of Matter and Center for Free-Electron Laser Science, Luruper Chaussee 149, Hamburg 22761, Germany; ‡ Department of Physics, Arizona State University, Tempe, Arizona 85287, United States; § Department of Chemistry, 6572 University of Pennsylvania, Philadelphia, Pennsylvania 19104, United States; ∥ College of Integrative Sciences and Arts, Arizona State University, Mesa, Arizona 85212, United States; ⊥ Initiative for Computational Catalysis (ICC), Flatiron Institute, Simons Foundation, 162 Fifth Avenue, New York, New York 10010, United States

**Keywords:** Polaritonic chemistry, optical cavity, electromagnetism, Maxwell’s equations, Ehrenfest dynamics

## Abstract

Molecular polaritons arise from electronic or vibrational strong coupling (ESC, VSC) with confined electromagnetic fields. While these have been widely studied, the influence of electron–nuclear dynamics in driven cavities remains largely unknown. Here, we report a previously unrecognized mechanism of vibrational activation that emerges under collective ESC in driven optical cavities. Using simulations that self-consistently combine Maxwell’s equations with quantum molecular dynamics, we show that collective electronic Rabi oscillations coherently drive nuclear motion. This effect is captured using both vibrational wave packet dynamics in a minimal two-level model and atomistic simulations based on time-dependent density-functional tight-binding theory. Vibrational activation depends nonmonotonically on the Rabi frequency and is maximized when the collective polaritonic splitting resonates with a molecular vibrational mode. The mechanism exhibits features consistent with a stimulated Raman-like relaxation mechanism. Our predictions are robust under realistic cavity conditions and provide the conditions in which they could be verified experimentally.

## Graphical abstract

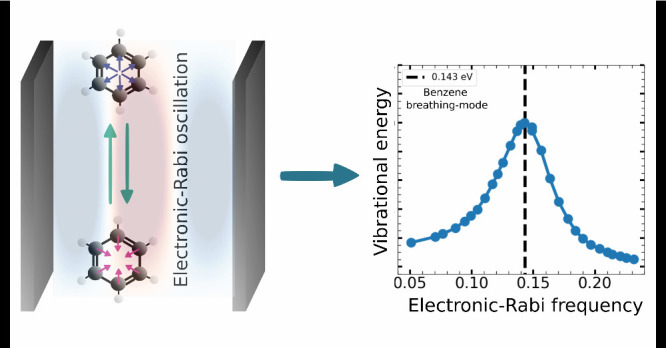

Inside optical cavities, molecules can interact strongly with confined electromagnetic modes, forming hybrid light–matter states that can modify molecular properties, a phenomenon underlying polaritonic chemistry.^1–12^ However, not all cavity-induced effects involve real photons or excited-state hybridization, which we refer to as *polaritons*. We therefore adopt the term *endyons* for polaron-like quasiparticles arising from vacuum electromagnetic fluctuations that induce static, zero-point renormalizations,^13^ distinguishing them from polaritons. This distinction is essential, as the present work focuses on the nonequilibrium dynamics of *molecular polaritons* rather than on ground-state reactivity in “dark” cavities.

Strong light–matter coupling can involve either electronic or vibrational molecular transitions. Electronic strong coupling (ESC) enables control of excited-state dynamics and photochemical pathways.^14–23^ Vibrational strong coupling (VSC), by contrast, involves infrared-active modes and has been widely explored for its potential to modify ground-state chemistry without external driving.^24–29^ Most theoretical studies of vibro-polaritons rely on *ab initio* ground-state calculations to predict cavity-modified vibrational spectra,^30–32^ limiting their ability to describe driven vibronic dynamics and leaving the interplay between ESC, cavity fields, and nuclear motion largely unexplored.

In this work, we demonstrate that individual molecular vibrational modes can be coherently activated inside driven optical cavities through ESC and that this activation is governed by the Rabi oscillations of the coupled light–matter system. By studying realistic cavity geometries, we establish a direct connection between electronic polaritonic dynamics and nuclear motion, revealing a mechanism for selectively driving vibrations under nonequilibrium conditions. To uncover this effect, we employ semiclassical simulations that self-consistently combine Maxwell’s equations for the cavity electromagnetic fields with quantum descriptions of molecular electron dynamics.^33,34^ As a minimal model, we first adopt the Born–Oppenheimer (BO) approximation and represent the molecules using two potential energy surfaces on which we propagate coupled vibrational wavepackets. This approach, termed throughout the text the two-level model, provides a description of the vibrational response in the presence of electronic Rabi oscillations.

To treat realistic polyatomic molecules, we further use a multiscale framework combining finite-difference time-domain (FDTD) solutions of Maxwell’s equations with atomistic electronic dynamics at the density functional tight-binding level.^33^ In this implementation, the electronic density matrix of each molecule is propagated self-consistently using the DFTB+ package,^35^ while nuclear motion is treated classically within the Ehrenfest approximation.^36^ Maxwell’s equations are solved in one and two spatial dimensions (see Methods section), whereas the molecular systems are described in three full dimensions, allowing a realistic representation of all cavity modes, spatial field profiles, and metal mirrors with a frequency-dependent dielectric response.^37^

Despite the known limitations of Ehrenfest dynamics for nuclear motion,^38^ we show that this level of theory captures the essential features of the driven vibrational activation induced by ESC. The results point to a stimulated Raman-like process driven by the interplay of upper and lower polaritonic fields as the underlying mechanism. From the perspective of the electromagnetic response, this behavior is consistent with polariton relaxation mediated by phonon emission. The vibrational driving frequency is determined by the collective Rabi splitting of the molecular ensemble rather than single-molecule coupling, and the efficiency and selectivity of the process depend on the cavity mode structure and quality factor. This collective character places the effect squarely within the realm of molecular polariton physics and highlights the role of cavity mediated intermolecular interactions, and cavity modified electronic interaction, in shaping nuclear dynamics.^39–41^

## Rabi-Driven Vibrational Activation via Polaritonic Resonance

We describe coupled electronic–nuclear dynamics within the Born–Oppenheimer (BO) approximation using two potential energy surfaces along a nuclear coordinate (Figure 1A), taken as the bond length of a diatomic molecule.

**Figure 1:**
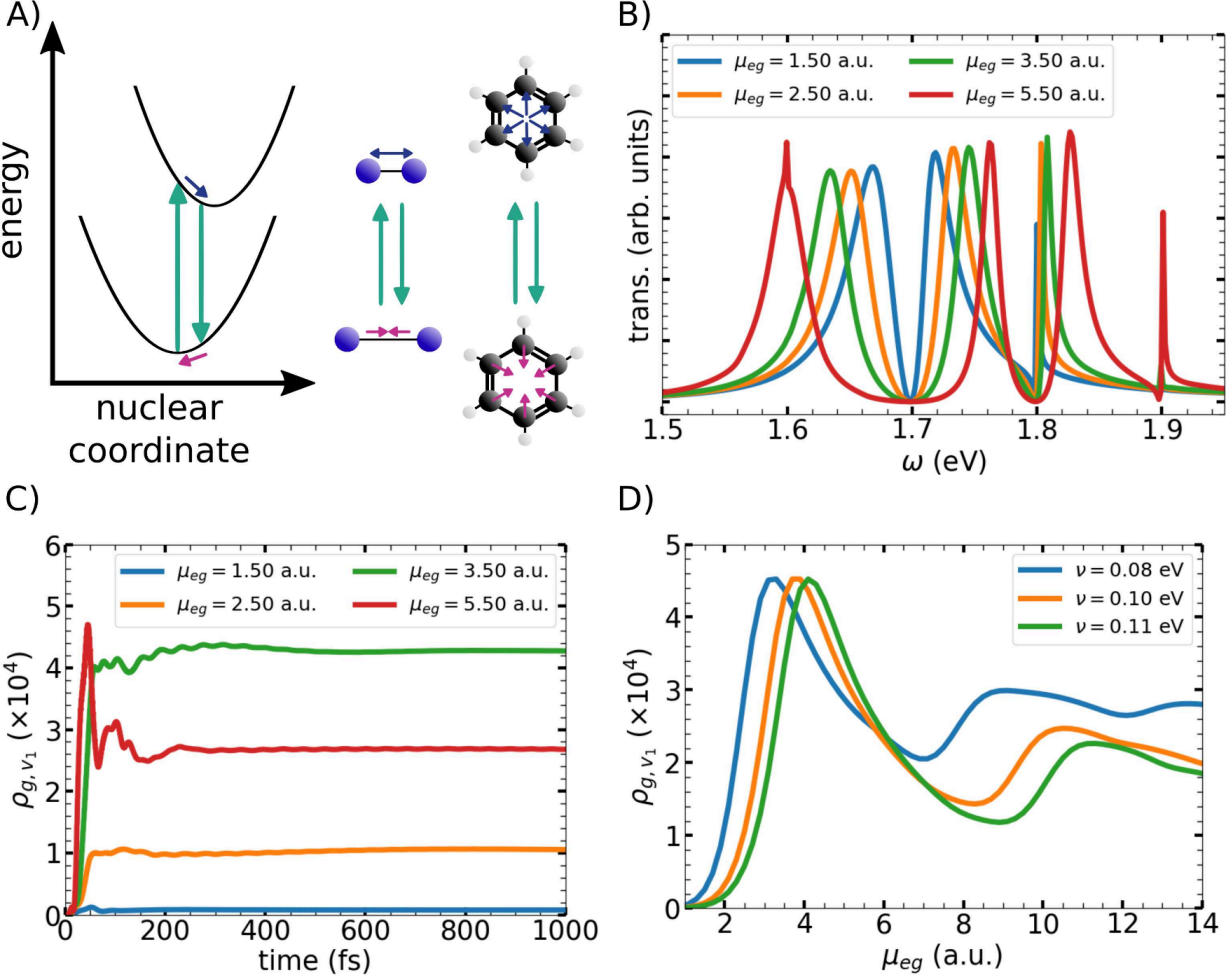
A) Schematic illustration of vibrational excitation induced by electronic strong coupling (ESC). The two parabolic curves represent the ground- and excited-state potential energy surfaces of a generic molecule. Cyan arrows indicate electronic absorption and emission processes, while blue and pink arrows denote the associated nuclear displacements during electronic excitation and relaxation. A diatomic molecule and a benzene molecule are shown as representative examples. B) Calculated transmission spectra for the two-level model as the effective electronic transition dipole moment μ_eg_ is varied, illustrating the evolution of the polaritonic response under increasing light–matter coupling. The spectra show the LP and UP peaks below and above 1.7 eV, respectively. Due to the quantum vibrational degrees of freedom, the UP peak is split.^42^ C) Time evolution of the occupation of the first vibrational state *v*_1_ of the electronic ground state for the two-level model with vibrational frequency ν = 0.1, eV. D) Steady-state occupation of the first vibrational state *v*_1_ of the electronic ground state as a function of μ_eg_ for three different vibrational frequencies. For clarity, the blue and green curves are normalized to the maximum of the orange curve.

To simulate the dynamics of this system under ESC, we propagate Schrödinger’s equation using the Hamiltonian defined in eq 6 of the Methods section, self-consistently coupled to one-dimensional Maxwell’s equations. The system consists of a realistic Fabry–Perót (FP) cavity formed by two 40 nm-thick gold mirrors with a Drude–Lorentz dielectric response, separated by 305.5 nm. An ensemble of molecules is placed at the center of the cavity on individual grid points, spanning a region 20 nm thick. The molecular electronic transition is tuned to be in resonance with the first cavity mode. The strength of the light–matter coupling is controlled by varying the electronic transition dipole moment μ_*eg*_. Additional details of the simulation setup are provided in the Methods section (Two Level Model with Vibrations).

Following excitation by a 5 fs pump pulse, ESC induces repeated absorption and emission, producing Rabi oscillations until cavity losses dissipate the field. The characteristic frequency of this process, termed the Rabi frequency (Ω), can be extracted from the transmission spectra by measuring the separation between the upper polariton (UP) and lower polariton (LP) peaks. When vibrational degrees of freedom are included, the UP peak splits into multiple vibro-polaritonic features following Franck–Condon selection rules (Figure 1B).^42^ This prevents the assignment of a single well-defined Rabi frequency. Nevertheless, the overall increase in the effective Rabi splitting with increasing μ_eg_ remains evident from the growing LP-UP separation.

Electronic Rabi oscillations induce vibrational activation, which we track by the time evolution of the occupation of the first vibrational state (*v*_1_) of the electronic ground state, as shown in Figure 1C. Following an initial transient, the occupation of this vibrational state reaches a steady value once optical energy is dissipated by cavity losses. This final occupation depends on μ_eg_ and, thus, on the effective Rabi frequency. Because the vibrational mode is optically inactive, further relaxation is inefficient.

Plotting the steady-state vibrational population versus μ_eg_ reveals a pronounced maximum (Figure 1D). We attribute this feature to a resonance between the vibrational frequency of the ground state and the Rabi frequency associated with ESC. Consistent with this interpretation, increasing the vibrational frequency ν, shifts the resonance to larger μ_eg_.

From a semiclassical perspective, this behavior can be understood as a driven damped harmonic oscillator, where the driving force arises from the time-dependent excited-state electronic density, analogous to displacive excitation of coherent phonons.^43^ The periodicity of this force is set by the electronic Rabi oscillations, whose frequency depends on the light–matter coupling strength. Energy transfer to the vibrational degree of freedom is maximized when the driving frequency resonates with the molecular vibrational frequency, resulting in a maximum occupation of the first vibrational state.

In addition to the dominant resonance, Figure 1D reveals a secondary maximum at larger values of the electronic transition dipole moment. Analysis of the underlying vibrational populations indicates that this feature originates from a Raman-type transition between the *v* = 0 and *v* = 2 vibrational states of the electronic ground state. A detailed investigation of this higher-order process and its dependence on system parameters lies beyond the scope of the present work.

For the semiclassical approximation, the damping leads to a broadening of the resonant response. In the present simulations, losses at the cavity mirrors effectively introduce damping into the system. Other electron–phonon dephasing mechanisms are not considered here, but they would not alter the conclusions presented below. Within this framework, shorter cavity lifetimes, corresponding to stronger damping, are expected to produce vibrational activation over a broader range of values of μ_eg_ or, equivalently, Rabi frequencies. The two-level model captures this behavior, which shows broader, less defined resonances with increasing mirror losses (Supplementary Figure 1).

Increasing the size of the molecular slab shifts the resonance to lower μ_eg_ values (Supplementary Figure 2) and reduces the population at resonance. While this may suggest a dilution of vibrational activation in large ensembles, such a conclusion is premature and requires further investigation.

A final feature of the Rabi-driven vibrational activation is that, under resonant conditions, the vibrational population scales with the fourth power of the driving field amplitude (Supplementary Figure 3), consistent with a Raman-like excitation mechanism (see Discussion).

## Rabi-Driven Vibrational Activation in Benzene within a Spatially Structured Cavity

We extend the analysis to polyatomic molecules in an FP cavity using Ehrenfest dynamics to describe coupled electronic and nuclear motion. We begin the study with benzene, whose first optically allowed π → π* excitation weakens the C–C bonds and leads to an expanded excited state ring geometry.^44^

Under resonant cavity conditions and following excitation, light–matter energy exchange induces a periodic contraction and expansion of the benzene ring, analogous to the diatomic molecule case (Figure 1A). Our simulations employ a one-dimensional Maxwell solver with two 50 nm aluminum mirrors (Drude–Lorentz dielectric response^37^) separated by 298 nm. The third cavity mode is tuned to the first electronic transition of benzene at 6.79 eV, predicted by DFTB and consistent with TDDFT.^45^

An ensemble of 201 benzene molecules (each lying in the *xy* plane) is placed over a 200 nm region at the cavity center to probe spatial effects. Further details of the simulation setup are provided in the Method section (DFTB systems). The strength of the light–matter coupling is varied by changing the effective molecular concentration parameter *N*_M_ in eq 5, from 1.69 × 10^–3^ nm^–3^ to 3.74 × 10^–2^ nm^–3^ (molar concentrations of 0.0028 and 0.0621 M).

Vibrational activation is quantified by the time-averaged vibrational potential energy (VPE) of each normal mode, obtained by projecting nuclear displacements onto normal modes and averaging after cavity energy dissipation; for more details, see Methods section (Average Vibrational Potential Energy).

The VPE is plotted as a function of the vibrational frequencies of the benzene molecule in Figure 2A, yielding a representation analogous to a vibrational spectrum for different values of the Rabi frequency. Owing to the classical treatment of the nuclear motion, the Rabi frequency can be determined unambiguously from the LP-UP splitting in transmission spectra (Supplementary Figure 4).

**Figure 2:**
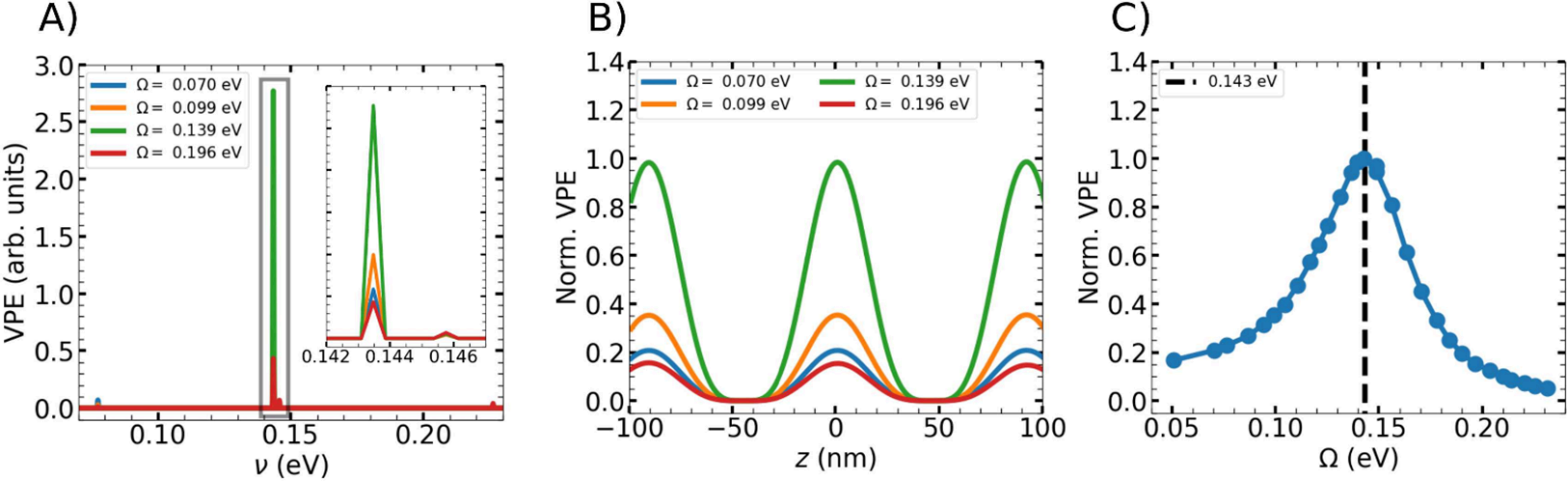
A) Vibrational potential energy (VPE) per normal mode of the central benzene molecule (*z* = 0.0, nm), plotted as a function of vibrational frequency and averaged over the last 100 fs of the simulation for different values of the Rabi splitting. The inset provides a magnified view of the highlighted frequency range. B) VPE of the benzene breathing mode (ν = 0.143, eV) for each molecule inside the cavity, shown as a function of molecular position for different values of the Rabi splitting. The VPE maps out the spatial profile of the third cavity mode, i.e., with two zeros at about ± 49 nm. C) Normalized VPE of the benzene breathing mode of the central molecule as a function of the Rabi splitting extracted from the transmission spectra, obtained by varying the molecular density parameter *N*_M_.

Only the breathing mode (0.143 eV) shows strong activation, while others show weak or no response. Its nuclear dynamics closely follows the behavior described previously and illustrated schematically in Figure 1A. This selectivity reflects both the frequency mismatch between the other modes and the applied Rabi splittings as well as the small projection of Rabi-induced nuclear motion onto them. Symmetry arguments may further rationalize this behavior^46^ and will be explored in future work.

As demonstrated in our previous work,^33^ the interaction between cavity modes and molecules depends strongly on the spatial position of the molecule due to the structure of the electromagnetic field. This spatial dependence is directly reflected in the Rabi-driven vibrational activation. As shown in Figure 2B, the breathing-mode VPE is maximized near the field antinode regions and suppressed near nodes.

The resonant character of the Rabi-driven vibrational activation is further illustrated in Figure 2C, where the VPE of the breathing mode shows a maximum when the Rabi frequency matches the vibrational frequency, corresponding to the condition Ω = ν. The Supplementary Movie visualizes the corresponding field and nuclear and electronic dynamics of the central molecule under resonant conditions. Tuning away from resonance progressively suppresses the vibrational activation, and no higher-order processes predicted by the two-level model are observed within the parameter range explored here.

Extending the analysis to two-dimensional cavities (Supplementary Figure 5A) increases losses, reducing mode selectivity. In benzene, this leads to simultaneous activation of multiple modes for a given Rabi splitting (Supplementary Figure 5B), broader resonances, and smaller VPE variations (Supplementary Figure 5C). Thus, it is shown that Rabi-driven vibrational activation is robust to cavity dimensionality, but vibrational mode selectivity requires high-quality cavities, potentially achievable by tailoring the cavity geometry using inverse design strategies.^34^

## Rabi-Driven Multimode Vibrational Activation

In previous examples, Rabi-driven nuclear motion projects mainly onto a single vibrational mode. This behavior, however, should not be regarded as universal. In more complex molecular systems, the electronic excitation can couple to multiple degrees of freedom. To illustrate such situations, we consider the pentacene molecule, which provides a suitable balance between structural complexity and analytical tractability due to its symmetry.

For these simulations, we employ a one-dimensional cavity geometry consisting of two 50 nm aluminum mirrors with a Drude–Lorentz dielectric response,^37^ separated by 180 nm. The fundamental cavity mode is resonant with the first electronic transition of pentacene at 3.975 eV. An ensemble of 101 pentacene molecules is arranged in the *xy* plane within a 100 nm region at the cavity center, with their molecular long axes aligned along the cavity polarization *x*. Additional details of the simulation setup are provided in the Methods section. Again, the light–matter coupling is controlled by varying the molecular density parameter *N*_M_ over the range 3.37 × 10^–4^ nm^–3^ to 3.37 × 10^–2^ nm^–3^ (molar concentrations of 5.6 × 10^–4^ M and 0.056 M).

Figure 3A reveals the simultaneous activation of multiple vibrational modes with their corresponding VPE exhibiting a clear dependence on the Rabi frequency. We focus on the ten most responsive modes, whose displacement patterns and frequencies are shown in Supplementary Figure 6. As in the diatomic and benzene cases, these modes are optically inactive and involve in-plane nuclear motion, consistent with the molecular orientation relative to the cavity field. They are characterized by C–C bond distortions, reflecting the weakening of multiple backbone C–C bonds in the electronically excited state that couples to the cavity and agrees with the geometrical selectivity discussed previously. By analyzing the VPE of each vibrational mode as a function of the Rabi frequency, we again observe a Rabi-resonant behavior for different vibrational modes. Each mode’s energy is maximized when it is in resonance with the Rab splitting, as shown in Figure 3B and C, where we present the results for the most sensitive modes in the range of Rabi frequencies considered in this work. Importantly, the orthogonality of the normal modes ensures that each vibrational mode is activated independently, provided that the nuclear dynamics in the cavity can be expressed in the normal-mode basis. As discussed above, the peak broadening depends on the cavity quality, while the peak height is determined by the electron–nuclear interaction and the mode’s contribution to the cavity nuclear dynamics.

**Figure 3:**
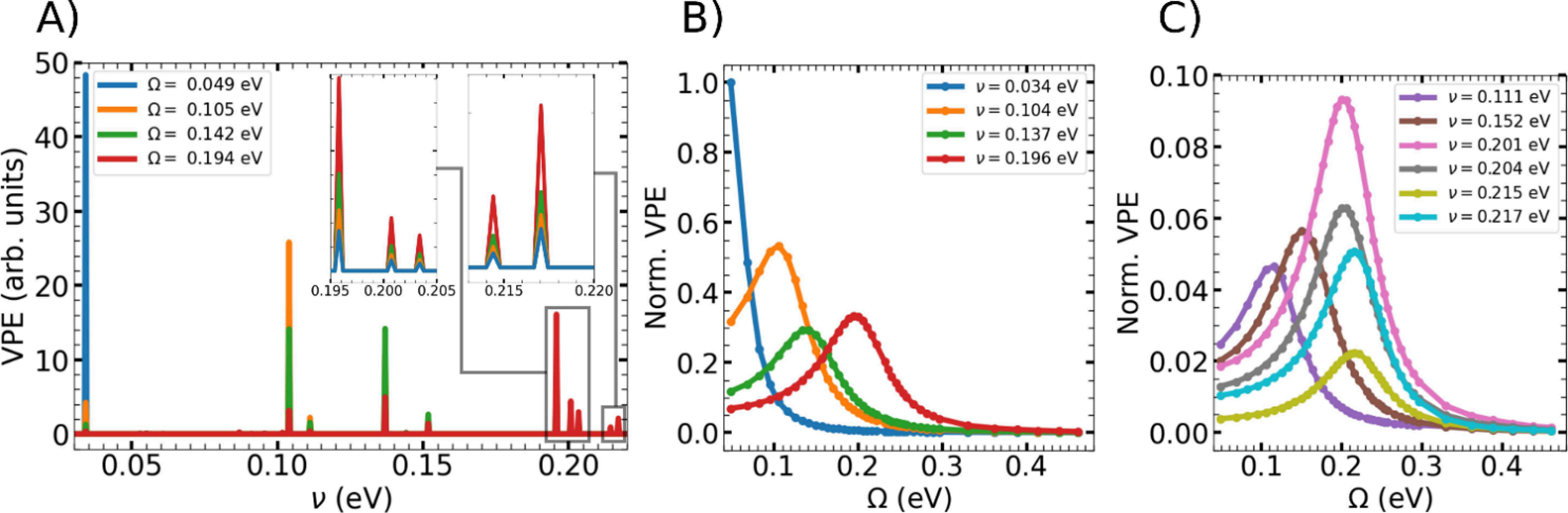
A) VPE of the central pentacene molecule plotted as a function of vibrational frequency, averaged over the final 100 fs of the simulation for different values of the Rabi frequency. The insets provide magnified views of the highlighted frequency ranges. Panels B) and C) show the normalized VPE of the most strongly affected vibrational modes of the central pentacene molecule plotted as a function of the Rabi frequency. Both panels share the same axis scale.

## Discussion

We identified a previously unreported mechanism of cavity-mediated vibrational excitation, which we term Rabi-driven vibrational activation, occurring when molecular ensembles under ESC are driven out of equilibrium. Despite its similarity to previous studies where vibrational activation occurs under VSC conditions,^47,48^ we observed that the presented phenomenon exhibits a pronounced maximum when the electronic Rabi frequency resonates with a molecular vibrational frequency, showing a pronounced maximum when the electronic Rabi frequency resonates with a molecular vibrational frequency. Nuclear Ehrenfest dynamics captures this behavior, establishing the Maxwell–DFTB framework as a versatile and predictive approach for studying Rabi-driven vibrational activation in polyatomic molecules. Using benzene and pentacene, we demonstrate that the process is mode-selective and governed by the collective nuclear response to a cavity-mediated electronic dynamic. When multiple modes are activated, their orthogonality ensures that each mode is driven independently once its resonance condition is satisfied. Increasing cavity losses broaden lineshapes so that an exact resonance between the Rabi frequency and the vibrational frequency is somewhat relaxed, enabling the activation of additional vibrational modes. Other sources of broadening, such as disorder, may also affect the process discussed here and deserve to be studied separately.

From a classical perspective, the mechanism can be rationalized in terms of a stimulated Raman-like process. In conventional stimulated Raman scattering (SRS), efficient vibrational excitation occurs when the frequency difference between pump and Stokes pulses matches a vibrational frequency (upper panel of Figure 4), with the vibrational amplitude scaling with the product of the field amplitudes.^49^

**Figure 4:**
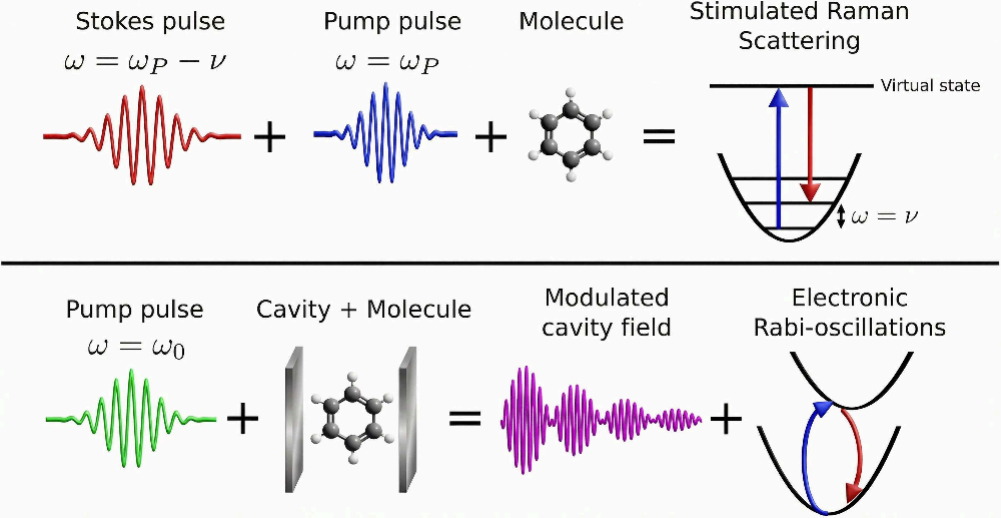
Upper panel illustrates the pump and Stokes pulses, together with their respective frequencies, required to induce stimulated Raman scattering (SRS). In this process, the molecule is promoted to a virtual electronic state, and the subsequent transition to the first vibrational level of the electronic ground state is driven by the Stokes pulse. The lower panel shows the resulting modulated intracavity electromagnetic field and the associated electronic Rabi oscillations that arise following excitation of the coupled cavity–molecule system, under resonant conditions where the cavity mode and the molecular electronic transition satisfy ω = ω_0_.

In the present simulations, the molecular system is driven by a temporally modulated intracavity field formed by the superposition of the UP and LP fields (bottom panel of Figure 4). A classical analysis (Supplementary Discussion 1) yields an analogous dependence on the product of polaritonic field components, closely mirroring the SRS.

Unlike conventional SRS, the effective “pump” and “Stokes” fields emerge self-consistently from collective strong light–matter coupling when the system is driven by a short pulse rather than two independent laser sources. In addition, the intracavity excitation leads to multiple cycles of absorption and emission during the cavity lifetime, in contrast to the single absorption–emission event characteristic of standard SRS (Figure 4).

An additional SRS-like signature of the Rabi-driven vibrational activation is the quadratic dependence of the population of the *v*_1_ vibrational state of the electronic ground state on the electric field intensity (Supplementary Figure 3), indicative of a second-order nonlinear process (Supplementary Discussion 1). These parallels with Raman scattering provide a rationale for the success of Ehrenfest dynamics in capturing the phenomenon, as this approach has been previously employed to describe Raman spectra and related vibrational processes.^36,50^

From a quantum-electrodynamic perspective, the process corresponds to UP → LP relaxation via phonon emission, thereby inducing vibrational activation. This interpretation is supported by calculations based on the Holstein–Tavis–Cummings (HTC) Hamiltonian (Supplementary Discussion 2), which provide direct access to the UP decay dynamics and the associated phonon generation (Supplementary Figure 7). In particular, phonon emission is maximized when the Rabi splitting between the UP and LP matches the vibrational frequency.

The semiclassical simulations reproduce this behavior under long-pulse excitation resonant with UP, although efficient phonon emission in this case still requires the presence of the LP field. As a result, mirror losses and laser line width play a critical role in enabling the UP → LP relaxation pathway. Broader cavity modes facilitate LP activation, as does a broader laser spectrum. In contrast, in the limiting case of ideal mirrors or an ultranarrow laser line width, phonon emission is fully suppressed. Notably, the HTC model predicts a UP → LP relaxation rate linear with laser intensity, assuming that the initial UP population scales linearly with the laser intensity. This behavior does not reproduce the quadratic dependence of the *v*_1_ ground-state population on the electric field intensity observed in the semiclassical simulations.

The HTC model further predicts a pronounced suppression of vibrational energy when the Rabi frequency exceeds the vibrational frequency (Supplementary Figure 8A), in agreement with the behavior observed in our Ehrenfest-based dynamics. At higher Rabi frequencies, a second maximum appears when the Rabi splitting is twice the vibrational frequency (Supplementary Figure 8B). This feature originates from a two-step relaxation pathway involving an intermediate dark state, followed by relaxation to the LP. Although this second peak resembles the secondary maximum observed in the two-level model results (Figure 1D), the semiclassical formulation does not explicitly resolve dark states and, therefore, cannot capture this quantum mechanism at a microscopic level. A detailed analysis of the origin of higher-order peaks in the vibrational populations predicted by the two-level model will be addressed in future work.

Finally, we suggest that this phenomenon could be experimentally explored in gas-phase molecular systems embedded in FP cavities, where the pressure provides a convenient handle to tune the collective Rabi splitting. Alternatively, solid-state platforms based on J-aggregate slabs inside FP cavities offer additional control as the Rabi splitting can be adjusted by varying the slab thickness or by using angle-resolved excitation. We hope that these considerations will motivate future experimental efforts to investigate the mechanism proposed here.

## Methods

### Maxwell + Quantum Dynamics

A detailed description of our implementation combining the numerical solution of Maxwell’s equations with either nuclear wavepacket dynamics or atomistic electronic dynamics based on density functional tight binding (DFTB) can be found in refs 42, 51 and ref 33, respectively. Here, we summarize the key elements relevant to the present work.

We solve Maxwell’s equations for the electric field **E** and magnetic field **B** on a spatial grid using the finite-difference time-domain (FDTD) method,^52^

1 $$\begin{array}{ll} \frac{\partial B\left(r,t\right)}{\partial t} & =-\nabla \times E\left(r,t\right),\\ \frac{\partial E\left(r,t\right)}{\partial t} & =c_{0}^{2}\nabla \times B\left(r,t\right)-\frac{1}{\epsilon_{0}}J\left(r,t\right), \end{array}$$

where **J** is the total current density. Simulations are performed in one and two spatial dimensions. Open boundary conditions are implemented using the convolutional perfectly matched layer (CPML) method.^52^

The current density **J** includes contributions from both the cavity mirrors and molecular polarization. The optical response of the metallic mirrors is described using a multipole Drude–Lorentz dielectric function,

2 $$\varepsilon \left(\omega \right)=1-\frac{\Omega_{D}^{2}}{\omega^{2}-i\Gamma_{D}\omega }-\underset{n}{\sum }\frac{\Delta \varepsilon_{n}\omega_{p}^{2}}{\omega^{2}-\omega_{n}^{2}-i\Gamma_{n}\omega }$$

with material parameters taken from ref 37. Aluminum parameters are used over the energy range 0.01 to 10 eV and gold parameters over 0.2 to 5.0 eV, ensuring an accurate description of realistic cavity dispersion.

The dielectric response in eq 2 is implemented through auxiliary differential equations for the metal current densities,

3 $$\frac{\partial J_{D}\left(r,t\right)}{\partial t}+\Gamma_{D}J_{D}\left(r,t\right)=\varepsilon_{0}\Omega_{D}^{2}E\left(r,t\right)$$

4 $$\frac{\partial^{2}J_{n}\left(r,t\right)}{\partial t^{2}}+\Gamma_{n}\frac{\partial J_{n}\left(r,t\right)}{\partial t}+\omega_{n}^{2}J_{n}\left(r,t\right)=\varepsilon_{0}\Delta \varepsilon_{n}\omega_{p}^{2}\frac{\partial E\left(r,t\right)}{\partial t}$$

The molecular contribution to the current density is given by **J**_mol_ = *∂* **P**_mol_/*∂t*, where **P**_mol_ is the macroscopic molecular polarization. The polarization at the position **r**_A_ of molecule *A* is computed as

5 $$P\left(r_{A},t\right)=N_{M}\left\langle {\mathrm{\mu ̂}}_{A}\right\rangle$$

where *N*_M_ is the molecular number density and $\left\langle {\mathrm{\mu ̂}}_{A}\right\rangle$ is the expectation value of the molecular dipole moment.^42,51,53^ Each molecule is assigned to an individual grid point, forming an effective continuous macroscopic medium. The molecular dipole moments are obtained from the time propagation of the electronic density matrix.^33,36^

### Two-Level Model with Vibrations

As a minimal and exact solvable molecular model, we propagate the time-dependent Schrödinger equation using a two-level Hamiltonian coupled to nuclear motion,

6 $$Ĥ=\left[\begin{array}{ll} \mathrm{T̂} & 0\\ 0 & \mathrm{T̂} \end{array}\right]+\left[\begin{array}{ll} V_{g} & -\mu_{eg}E_{x}\\ -\mu_{eg}E_{x} & V_{e} \end{array}\right]$$

where T̂ is the nuclear kinetic energy operator, μ_eg_ is the electronic transition dipole moment, and *E*_*x*_ is the *x*-component of the electric field, corresponding to the polarization used in the one-dimensional simulations.

The ground- and excited-state potential energy surfaces are modeled as harmonic oscillators,

7 $$\begin{array}{ll} V_{g} & =\frac{1}{2}M\nu {\left(R-R_{0}\right)}^{2},\\ V_{e} & =\frac{1}{2}M\nu {\left(R-R_{0}-\Delta R\right)}^{2}+\Delta V, \end{array}$$

where *M* is the reduced mass, *R*_0_ is the ground-state equilibrium coordinate, Δ*R* is the shift between ground- and excited-state equilibria, and Δ*V* is the electronic excitation energy. We use parameters corresponding to an N_2_-like reduced mass, with Δ*V* = 1.6985 eV, Δ*R* = 0.023 au, and *R*_0_ = 0. Unless stated otherwise, the vibrational frequency is ν = 0.10 eV. Additional values ν = 0.08 and 0.11 eV are used for Figure 1.

The nuclear wavepacket dynamics is propagated using the split-operator method with a time step of 0.05 fs. Each molecule acts as an induced dipole under the local electric field, and its time derivative contributes to the Maxwell equations via eq 5. The molecular density is fixed at *N*_M_ = 7.0 × 10^–2^ nm^–3^. The system is excited by a short pulse with a peak amplitude of 0.5 V/nm and duration of 5 fs. FDTD grid spacings and time steps match those used in the DFTB simulations.

### DFTB Systems

Benzene and pentacene molecules are simulated using time-dependent density functional tight binding (TDDFTB) theory,^54,55^ as implemented in the DFTB+ package.^35,36^ Nuclear motion is treated at the Ehrenfest level.^36^ The mio-1-1 Slater–Koster parameter set is employed,^54^ and molecular geometries are optimized prior to the coupled Maxwell–TDDFTB simulations.

For benzene in one-dimensional cavities, we use a grid spacing Δ*z* = 1.0 nm, a Maxwell time step Δ*t*_Mxll_ = 2.419 × 10^–4^ fs, a molecular time step Δ*t*_mol_ = 5Δ*t*_Mxll_, and a total simulation time of 400 fs. The excitation pulse has a peak amplitude of 0.05 V/nm, a frequency of 7.0 eV, and a duration of 4.0 fs.

Pentacene simulations use a one-dimensional cavity with Δ*z* = 1.0 nm, identical time steps, and a total simulation time of 400 fs. The excitation pulse has a peak amplitude of 0.7 V/nm, a frequency of 4.0 eV, and a duration of 6.0 fs.

### Average Vibrational Potential Energy

To quantify vibrational activation, nuclear displacements are projected onto the molecular normal modes,^56,57^

8 $$Q_{i}\left(t\right)=\underset{A}{\sum }m_{A}\Delta r_{A}\left(t\right)\cdot v_{A,i}$$

where the sum runs over nuclei *A*, **v**_A,*i*_ are the normal mode eigenvectors, Δ**r**_A_ are displacements relative to equilibrium, and *m*_A_ are atomic masses.

The potential energy associated with mode *i* is then defined as

9 $$V_{i}\left(t\right)=\frac{1}{2}{\left(\nu_{i}Q_{i}\left(t\right)\right)}^{2}$$

where ν_*i*_ is the vibrational frequency of the mode. The average vibrational potential energy (VPE) is obtained by time averaging *V*_*i*_(*t*) over the final portion of the simulation after most of the optical energy has dissipated. For benzene and pentacene, averages are taken over the final 100 fs.

## Supplementary Material





## Notes

The following files are available free of charge. The Supporting Information is available free of charge at https://pubs.acs.org/doi/10.1021/acs.nanolett.6c00832.

Additional results and discussions supporting the main text, as well as a more detailed caption for the Supplementary Movie (PDF)
Supplementary movie: shows selected observables from Maxwell–DFTB dynamics of benzene molecules in a Fabry–Pérot cavity under electronic strong coupling (MP4)
