# Supplementary material for: Collective Rabi-Driven Vibrational Activation in Molecular Polaritons
Source: Nano Lett. 2026 Apr 7;26(15):5298–306. doi: 10.1021/acs.nanolett.6c00832 (PMC13107513; doi:10.1021/acs.nanolett.6c00832)
Supplement: Supplementary file 1 [file nl6c00832_si_001.pdf]

Supplementary Information for Collective Rabi-driven vibrational activation in molecular polaritons

Carlos M. Bustamante *[1], Franco P. Bonafé †[1], Richard Richardson[2], Michael Ruggenthaler[1], Wenxiang Ying[3], Abraham Nitzan ‡[3], Maxim Sukharev §[2,4], and Angel Rubio ¶[1,5]

[1]Max Planck Institute for the Structure and Dynamics of Matter and Center for Free-Electron Laser Science, Luruper Chaussee 149, Hamburg 22761, Germany
[2]Department of Physics, Arizona State University, Tempe, Arizona 85287, United States
[3]Department of Chemistry, University of Pennsylvania, Philadelphia, Pennsylvania 19104, United States
[4]College of Integrative Sciences and Arts, Arizona State University, Mesa, Arizona 85212, United States
[5]Initiative for Computational Catalysis (ICC), Flatiron Institute, Simons Foundation, 162 5th Avenue, New York, NY 10010 USA

Throughout this Supplementary Information, the symbol Ω denotes the Rabi splitting between the upper and lower polariton modes expressed in energy units, ν denotes molecular vibrational mode energies, and N_M denotes the effective molecular number density used to control the collective light–matter coupling strength.

Supplementary Results

Supplementary Figure 1 presents results obtained with the two-level model when different mirror thicknesses are considered. Decreasing the mirror thickness increases cavity losses. The final v_1 population, plotted versus μ_{eg},

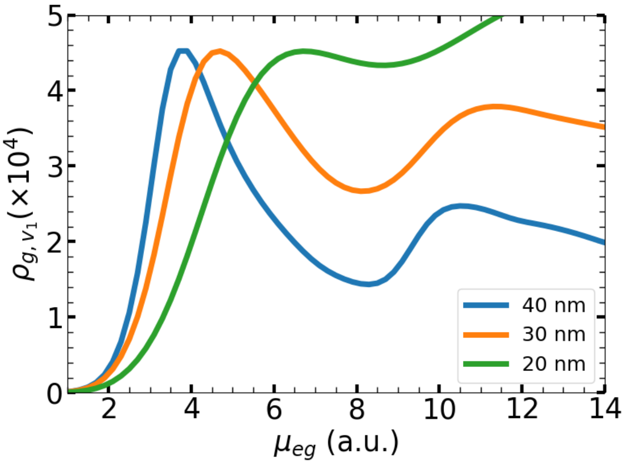

Supplementary Figure 1: Occupation of the first vibrational state v_1 of the electronic ground state at long times, plotted as a function of μ_{eg} for three mirror thicknesses.

still exhibits a pronounced maximum, but the resonance peak broadens as losses increase. Changes in mirror thickness also shift the cavity mode structure; to maintain resonance with the molecule we adjusted molecular parameters accordingly, which in turn required different values of μ_{eg} to reach resonance.

*carlos.bustamante@mpsd.mpg.de
†franco.bonafe@mpsd.mpg.de
‡anitzan@sas.upenn.edu
§Maxim.Sukharev@asu.edu
¶angel.rubio@mpsd.mpg.de

Supplementary Figure 2 shows that, for larger molecular slab sizes, both the value of μ_{eg} required to reach the resonant condition and the maximum vibrational population are reduced.

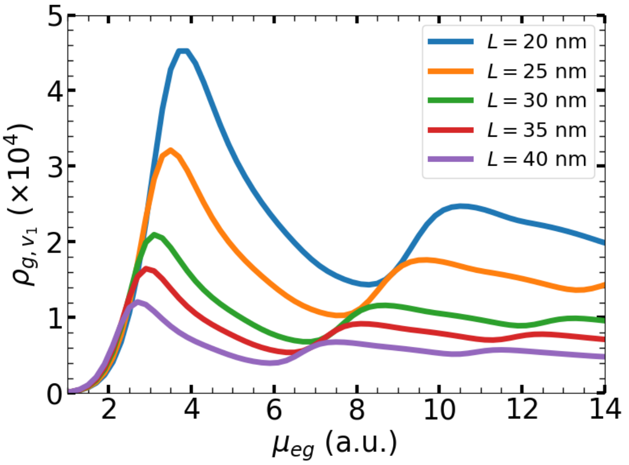

Supplementary Figure 2: Occupation of the first vibrational state v_1 of the electronic ground state at long times, plotted as a function of μ_{eg} for different molecular slab sizes (L).

Supplementary Figure 3 shows the vibrational population at resonance as a function of the applied electric field amplitude. The fit indicates $\rho_{g,v_1} \propto |E_x|^4$, equivalent to ρ_{g,v_1} scaling with the square of the laser intensity, consistent with a second-order process.

Supplementary Figure 4 shows transmission spectra from Ehrenfest simulations of the benzene ensemble described in the main text. The two polaritonic peaks are readily identified, allowing extraction of the Rabi splitting Ω and the corresponding spectral Rabi frequency.

Supplementary Figure 5 shows the results of the extended analysis of Rabi-driven vibrational activation to two-dimensional. The simulation box spans 340×340 nm^2 in the xy plane with grid spacing $\Delta x = \Delta y = 1$ nm. A 20 nm boundary region is assigned to the CPML. Time steps and total simulation time are identical to the one-dimensional case. The system is excited by a point-like pulse applied at the cavity center with frequency 7.0 eV and a Gaussian envelope of 1.5 fs full width at half maximum. In this case, we consider a ring cavity geometry, as illustrated in Supplementary Fig. 5A, with an inner radius of 56 nm and an outer radius of 106 nm. The fundamental cavity mode is tuned to resonance with the first electronic transition of the benzene molecule. As in the one-dimensional case, the optical response of the cavity mirrors is described using Drude–Lorentz parameters for aluminium. A total of 221 benzene molecules are placed at the center of the cavity on the circumference of a circle with a radius of 8 nm, with each molecule occupying an individual grid point, as shown in the inset of Supplementary Fig. 5A. The strength of the light–matter coupling is controlled by varying the molecular density parameter N_M between 0.067 nm^{-3} and 0.337 nm^{-3}, corresponding to molar concentrations of 0.11 M and 0.56 M, respectively.

Supplementary Fig. 5B shows how the higher losses introduced by the additional spatial dimension decrease the specificity of Rabi-driven vibrational activation, leading to the concurrent activation of multiple vibrational modes for a given Rabi splitting.

Despite the increased broadening of the resonant response due to these losses, the hallmark of Rabi-driven vibrational activation remains clearly observable in the two-dimensional cavity, as shown in Supplementary Fig. 5C. In particular, the VPE of the benzene breathing mode retains a pronounced dependence on the Rabi frequency and exhibits a maximum under resonant conditions.

Supplementary Figure 6 displays the atomic-displacement patterns of the normal modes in pentacene that are most sensitive to the Rabi-driven effect discussed in Figure 4 of the main text. These modes are optically inactive by symmetry and are characterized by significant C–C displacements.

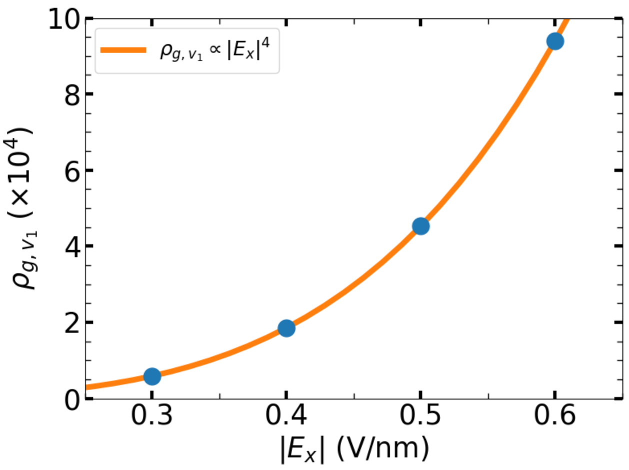

Supplementary Figure 3: Occupation of the first vibrational state v_1 of the ground state at resonance ($\nu = 0.1$ eV) as a function of the peak amplitude of the source electric field (blue dots). The orange curve is a fit showing a dependence $\rho_{g,v_1} \propto |E_x|^4$.

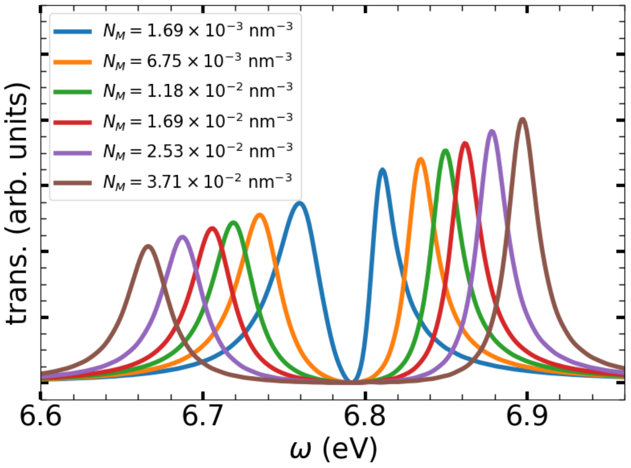

Supplementary Figure 4: Transmission spectra for 201 benzene molecules inside an optical cavity resonant with the first electronic transition, shown for different values of the density parameter N_M.

Supplementary Discussion 1: Classical forced oscillator / SRS analogy

A classical picture provides a transparent connection between the Rabi-driven activation described in the main text and a stimulated Raman-like mechanism. Consider a forced, damped harmonic oscillator describing a normal coordinate Q,

$$\ddot{Q} + \gamma \dot{Q} + \nu^2 Q = F_Q(t), \tag{S1}$$

where ν is the vibrational frequency, γ is a damping rate, and $F_Q(t)$ is the driving force. Assuming a Raman-like origin for the driving force,

$$F_Q(t) \approx \tfrac{1}{2} \frac{\partial \alpha}{\partial Q} |E(t)|^2, \tag{S2}$$

with α the dynamic polarizability and $E(t)$ the electric field. Writing the intracavity field as a superposition of lower- and upper-polariton components,

$$E(t) = E_{LP} e^{-\mathrm{i}\omega_{LP} t} + E_{UP} e^{-\mathrm{i}\omega_{UP} t} + \text{c.c.}, \tag{S3}$$

the intensity contains a beat term,

$$|E(t)|^2 \supset 2|E_{LP}||E_{UP}| \cos(\Omega t), \tag{S4}$$

where $\Omega = \omega_{UP} - \omega_{LP}$ is the Rabi splitting. (Other terms oscillate at optical frequencies and average out for $\nu \ll \omega_{LP}, \omega_{UP}$.)

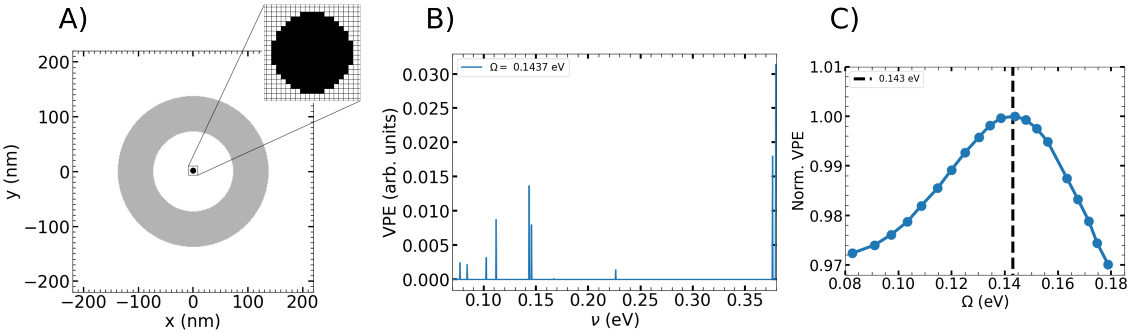

Supplementary Figure 5: A) Schematic representation of the ring cavity used in the two-dimensional simulations. The cavity has an inner radius of 56 nm and an outer radius of 106 nm. A total of 221 benzene molecules are placed at the center of the cavity, occupying a circular region with a radius of 8 nm. The inset highlights the grid points corresponding to the positions of individual molecules. B) VPE of the central benzene molecule, located at $\mathbf{r} = (0.0, \mathrm{nm}, 0.0, \mathrm{nm})$, plotted as a function of vibrational frequency and averaged over the final 100 fs of the simulation. C) Normalized VPE of the benzene breathing mode of the central molecule as a function of the Rabi splitting extracted from the transmission spectra, obtained by varying the molecular density parameter N_M.

Inserting the beat term into Eq. (S1) and solving for the long-time driven amplitude yields

$$Q_0 \propto |\chi_\nu(\Omega)|\,|E_{LP}|\,|E_{UP}|, \tag{S5}$$

with the susceptibility

$$\chi_\nu(\Omega) \propto \frac{1}{\nu^2 - \Omega^2 - \mathrm{i}\gamma\Omega}. \tag{S6}$$

Thus the driven amplitude is maximal when $\Omega \approx \nu$, reproducing the resonance condition observed in the simulations. The combination of Eqs. (S5) and (S6) parallels the dependence found in stimulated Raman scattering, with E_{LP} and E_{UP} playing roles analogous to pump and Stokes fields [1].

Assuming that only the vibrational states v_0 and v_1 are significantly populated and that $\rho_{v_1} \ll \rho_{v_0}$, one obtains $\langle Q\rangle \propto \sqrt{\rho_{v_1}}$. Combining this relation with Eq. (S5) explains the observed scaling $\rho_{g,v_1} \propto |E_x|^4$ reported in Supplementary Fig. 3.

Supplementary Discussion 2: Holstein–Tavis–Cummings analysis

We present here a Holstein–Tavis–Cummings (HTC) model [2, 3] to analyze Rabi-driven vibrational excitation from a quantum-electrodynamical perspective. The HTC model describes N electronic two-level systems, each coupled to a local harmonic vibration, and collectively coupled to a single cavity mode. The HTC analysis complements the mean-field Maxwell+Ehrenfest simulations: the latter capture spatially resolved macroscopic polarization and nonlinear response but neglect light–matter entanglement, while the HTC model resolves bright and dark manifolds and phonon-mediated relaxations in the single-excitation regime.

The HTC Hamiltonian reads

$$\hat{H}_{\mathrm{HTC}} = \hat{H}_{\mathrm{M}} + \hat{H}_{\mathrm{cav}} + \hat{H}_{\mathrm{LM}}, \tag{S7}$$

with

$$\hat{H}_{\mathrm{M}} = \sum_{n=1}^{N} \hbar\omega_0 \hat{\sigma}_n^+ \hat{\sigma}_n^- + \hbar\nu \sum_n \hat{b}_n^\dagger \hat{b}_n + \sum_n \hat{\sigma}_n^+ \hat{\sigma}_n^-\, c_\nu (\hat{b}_n + \hat{b}_n^\dagger), \tag{S8}$$

$\hat{H}_{\mathrm{cav}} = \hbar\omega_{\mathrm{c}}(\hat{a}^\dagger\hat{a} + \frac{1}{2})$, and $\hat{H}_{\mathrm{LM}} = \hbar g_{\mathrm{c}} \sum_{n=1}^{N} (\hat{a}^\dagger \hat{\sigma}_n^- + \hat{a}\hat{\sigma}_n^+)$. Here $\hat{\sigma}_n^\pm$ are excitonic raising/lowering operators, $\hat{b}_n$ are local phonon annihilation operators of frequency ν, and c_ν is the exciton–phonon coupling. The shift between ground- and excited-state minima is $\Delta R = \sqrt{2c_\nu^2/\nu^3}$ and the Huang–Rhys factor is $S = (c_\nu/\nu)^2$.

The purely electronic-photonic part forms the Tavis–Cummings (TC) Hamiltonian,

$$\hat{H}_{\mathrm{TC}} = \sum_{n=1}^{N} \hbar\omega_0 \hat{\sigma}_n^+ \hat{\sigma}_n^- + \hbar\omega_{\mathrm{c}} \left(\hat{a}^\dagger\hat{a} + \tfrac{1}{2}\right) + \hbar g_{\mathrm{c}} \sum_{n=1}^{N} (\hat{a}^\dagger \hat{\sigma}_n^- + \hat{a}\hat{\sigma}_n^+). \tag{S9}$$

In the single-excitation manifold one can define the collective bright state $|\mathrm{B}\rangle = \frac{1}{\sqrt{N}} \sum_n |\mathrm{E}_n, 0\rangle$ which couples to the one-photon state $|\mathrm{G}, 1\rangle$. Diagonalization yields the upper and lower polariton eigenstates $|\pm\rangle$

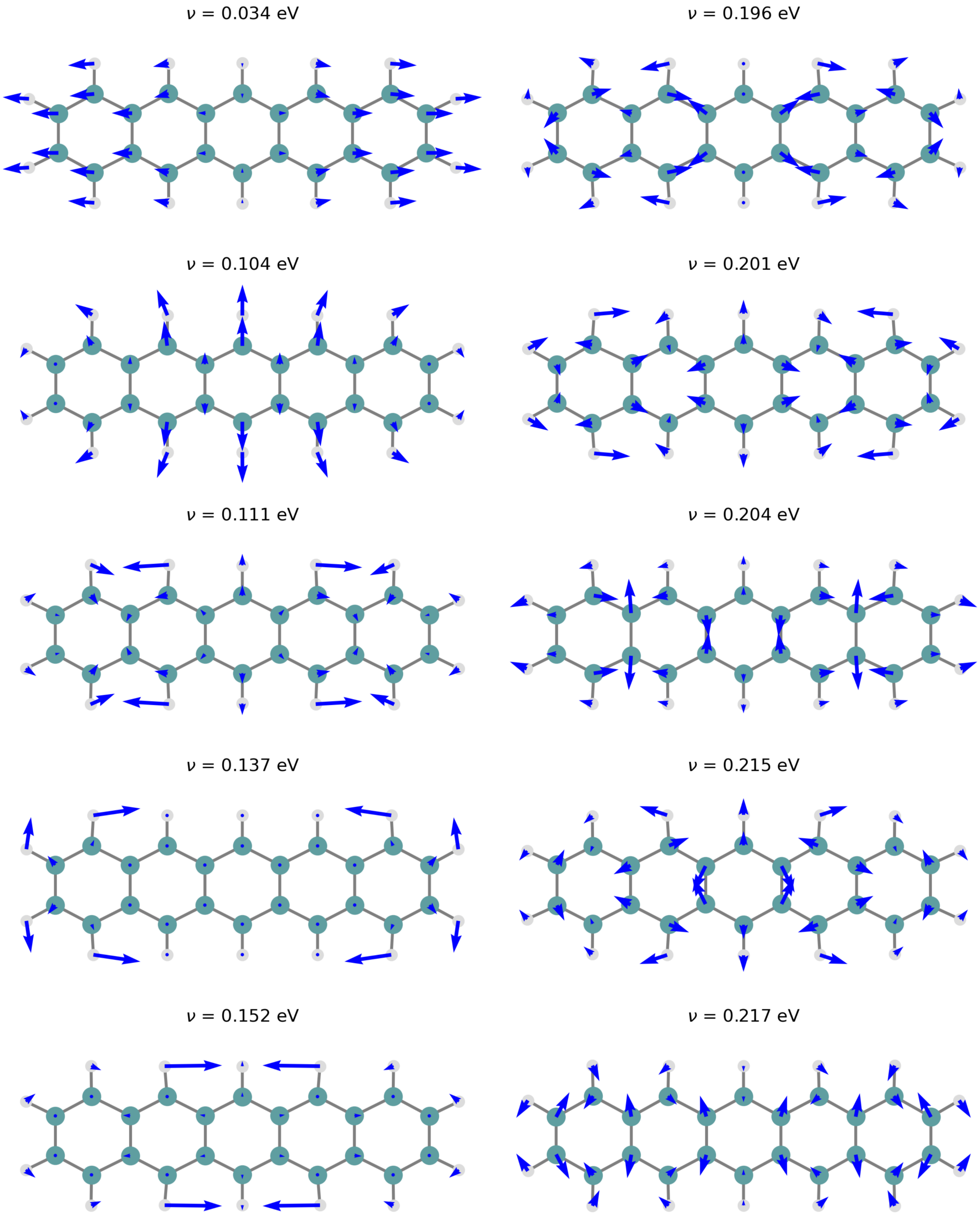

Supplementary Figure 6: Atomic displacement patterns for the ten normal modes most strongly affected by the Rabi oscillations, together with their vibrational frequencies.

with energies $\omega_\pm$. We define the collective Rabi splitting

$$\Omega \equiv \omega_+ - \omega_- = \sqrt{(\omega_c - \omega_0)^2 + 4Ng_c^2}, \tag{S10}$$

which reduces to $\Omega = 2\sqrt{N}g_c$ at resonance $\omega_c = \omega_0$.

Dark states $|D_k\rangle$ (with $k = 1, \ldots, N-1$) complete the single-excitation basis; their energies remain at the exciton site energy in the homogeneous model. Fourier transforming the phonon operators, $\hat{b}_k = \frac{1}{\sqrt{N}} \sum_n e^{2\pi i n k/N}\, \hat{b}_n$, and expressing the HTC Hamiltonian in the polariton basis yields exciton–phonon coupling terms that mediate transitions among $|+\rangle$, $|-\rangle$, and $|D_k\rangle$. In particular, transitions become resonant when energy gaps match phonon energies.

We solve the time-dependent Schrödinger equation,

$$\hat{H}_{\mathrm{HTC}}|\Psi(t)\rangle = i\hbar\frac{\partial}{\partial t}|\Psi(t)\rangle, \tag{S11}$$

within the single-excitation manifold using a finite vibrational Fock basis for each mode. Simulations are initialized with the system in the upper polariton and all vibrations in their ground state, $|\Psi(0)\rangle = |+\rangle \bigotimes_{n=1}^{N} |0_n\rangle$.

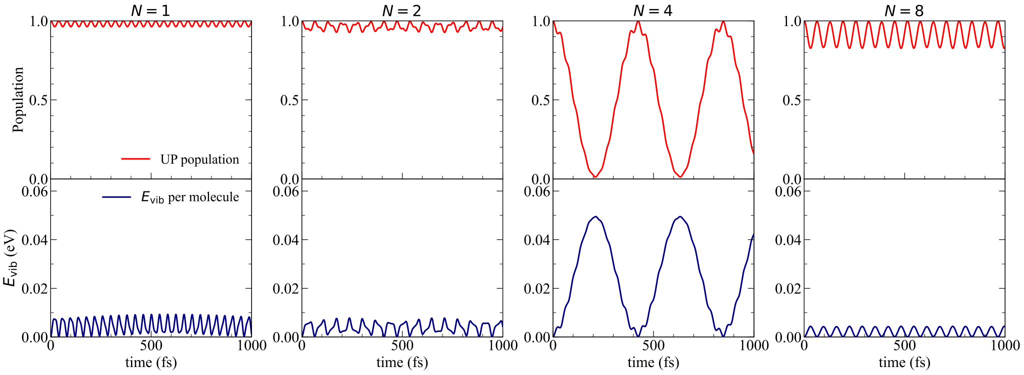

Supplementary Figure 7: Quantum dynamics of the UP population and vibrational energy per molecule for different numbers of molecules N.

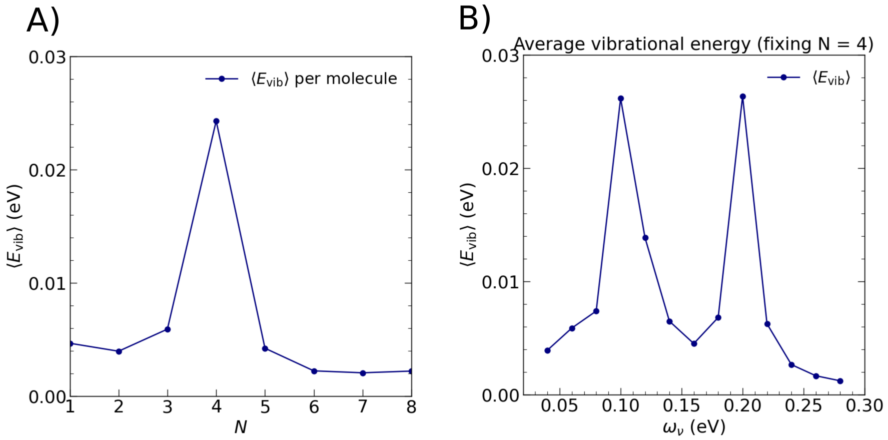

Supplementary Figure 8: Time-averaged vibrational energy per molecule. (A) $\langle E_{\mathrm{vib}} \rangle$ as a function of N for fixed $g_{\mathrm{c}} = 0.05$ eV and $\nu = 0.2$ eV, showing a maximum at resonance $N = 4$. (B) $\langle E_{\mathrm{vib}} \rangle$ as a function of ν for fixed $N = 4$ and $\Omega = 0.2$ eV, showing resonances at $\nu = \Omega/2$ and $\nu = \Omega$.

The dynamics are propagated with a Trotter integrator with time step $\Delta t = 0.025$ fs and total propagation $T = 1$ ps.

We monitor the UP population

$$P_{\mathrm{UP}}(t) = \langle \Psi(t)|+\rangle\langle+|\Psi(t)\rangle, \tag{S12}$$

and the vibrational energy per molecule

$$E_{\mathrm{vib}}(t) = \frac{\hbar\nu}{N}\sum_{n=1}^{N}\langle\Psi(t)|\hat{b}_n^\dagger\hat{b}_n|\Psi(t)\rangle. \tag{S13}$$

Supplementary Fig. 7 shows representative quantum dynamics of the UP population and vibrational energy per molecule for different values of N, with $g_{\mathrm{c}} = 0.05\,\mathrm{eV}$ and $\nu = 0.20\,\mathrm{eV}$. The resonance condition $\nu = \Omega$ is reached for $N = 4$ (since $\Omega = 2\sqrt{N}g_{\mathrm{c}}$ at resonance). Off resonance ($N = 1, 2, 8$) the UP population remains close to unity and the vibrational energy per molecule stays small, while at resonance ($N = 4$) UP population dynamics show pronounced oscillations accompanied by large-amplitude vibrational energy oscillations, indicating efficient polariton-to-phonon energy transfer.

To quantify vibrational excitation efficiency we compute the time-averaged vibrational energy per molecule,

$$\langle E_{\mathrm{vib}}\rangle = \frac{1}{T}\int_0^T dt\, E_{\mathrm{vib}}(t), \tag{S14}$$

with total propagation $T = 1$ ps.

Supplementary Fig. 8 summarizes $\langle E_{\mathrm{vib}}\rangle$ as a function of system parameters. Panel A fixes $g_{\mathrm{c}} = 0.05$ eV and $\nu = 0.2$ eV and scans N, revealing a clear maximum at $N = 4$ where $\nu = \Omega$. Panel B fixes $N = 4$ and $\Omega = 0.2$ eV and scans ν, revealing two pronounced resonances at $\nu = \Omega/2$ and $\nu = \Omega$, corresponding to UP $\rightarrow$ DS $\rightarrow$ LP (two-phonon) and UP $\rightarrow$ LP (one-phonon) relaxation channels, respectively.

Taken together, the Maxwell+Ehrenfest and the HTC descriptions identify the same polaritonic normal modes and vibronically induced resonances. In the QED picture, UP $\rightarrow$ LP + vibron(s) transitions describe polariton relaxation into phonons, whereas in the Maxwell+Ehrenfest picture the same physics appears as Raman-like frequency mixing between coupled field and polarization amplitudes. The essential formal distinction is that mean-field factorization in Maxwell+Ehrenfest eliminates light–matter entanglement and represents dark-state physics implicitly via spatially structured polarization modes, allowing for nonlinear molecular responses; the QED HTC model, by contrast, resolves bright and dark manifolds explicitly but is restricted to the single-excitation (linear-response) regime.

Caption for Supplementary Movie

The movie shows selected observables from Maxwell–DFTB dynamics of benzene molecules in a FP cavity under ESC, when the corresponding Rabi-frequency resonates with the benzene breathing mode. The left panel shows the time evolution of the electric field in the simulation box. The gray rectangles indicate the positions of the aluminum mirrors, and the cyan rectangle marks the region containing 201 benzene molecules. The dynamics is initiated by pumping the system with an ultrashort pulse incident on the cavity through the right mirror. The middle panel shows the nuclear displacement of the benzene molecule located at the center of the cavity. The displacement is amplified by a factor of 5000 to visually highlight the breathing-mode–like nuclear dynamics. The right panel shows the time evolution of the electronic energy of the benzene molecule at the center of the FP cavity.

References

[1] Robert W Boyd. “Chapter 10-Stimulated Raman scattering and stimulated Rayleigh-wing scattering”. In: *Nonlinear Optics* (2020), pp. 479–513.

[2] Michael Tavis and Frederick W Cummings. “Exact solution for an N-molecule—radiation-field Hamiltonian”. In: *Physical Review* 170.2 (1968), p. 379.

[3] Michael Tavis and Frederick W Cummings. “Approximate solutions for an N-molecule-radiation-field Hamiltonian”. In: *Physical Review* 188.2 (1969), p. 692.
